# Adrenal myelolipoma a rare benign tumour managed laparoscopically: Report of two cases

**DOI:** 10.4103/0972-9941.59312

**Published:** 2009

**Authors:** Bandar Al Harthi, Muhammad M Riaz, Amal H Al Khalaf, Mohammad Al Zoum, Wafa Al Shakweer

**Affiliations:** Department of Surgical Specialties, Breast and Endocrine Division, Riyadh, Saudi Arabia

**Keywords:** Angiomyelolipoma, laparoscopic adrenalectomy, myelolipoma

## Abstract

Adrenal Myelolipoma is a rare benign neoplasm composed of mature adipose tissue and a variable amount of haemopoietic elements. Most lesions are small and asymptomatic, discovered incidentally during autopsy or on imaging studies performed for other reasons. Two cases of myelolipoma are presented here, where the tumours were hormonally inactive, but presented with abdominal symptoms and were managed by laparoscopic adrenalectomy.

## INTRODUCTION

Myelolipomas are rare, benign tumours, usually found in the adrenal gland, although myelolipomatous foci can be present in other extra-adrenal locations. These tumours were initially described by Giercke in 1905, and 24 years later, Oberling coined the team ‘myelolipoma’. They are composed of mature adipocytes and normal haematopoietic tissue. Although they do not represent a hematopoietic source, they contain precursors of white and red blood cells such as megakaryocytes.[1]

The incidence of adrenal myelolipoma is reported as being 0.08 to 0.4% at autopsy.[2] More often than not, the male-to-female ratio is 1:1, however, a study reported a male-to-female ratio of 2:3.[3] With the vast use of non-invasive imaging its incidental detection has become more common, reaching up to 7% of the adrenal masses.[4] Adrenal myelolipoma present as a site of extramedullary haematopoiesis. Although great numbers of incidentally discovered lesions are small and asymptomatic, reports are not infrequent for cases of large symptomatic lesions. The most well-recognised complication of adrenal myelolipoma is spontaneous retroperitoneal haemorrhage.[5,6] No potential of malignancy for adrenal myelolipoma has been proved. Symptomatic lesions must be treated, and with the advent of minimal invasive surgery, laparoscopic adrenalectomy has shown a considerable decrease in surgically derived morbidity as well as hospital stay and convalescence.[7]

We report two cases of symptomatic myelolipoma where diagnosis was made on the basis of radiological features and image-guided fine-needle biopsies, and were treated with laparoscopic adrenalectomy.

## CASE REPORTS

### Case 1

**Figure 1:**
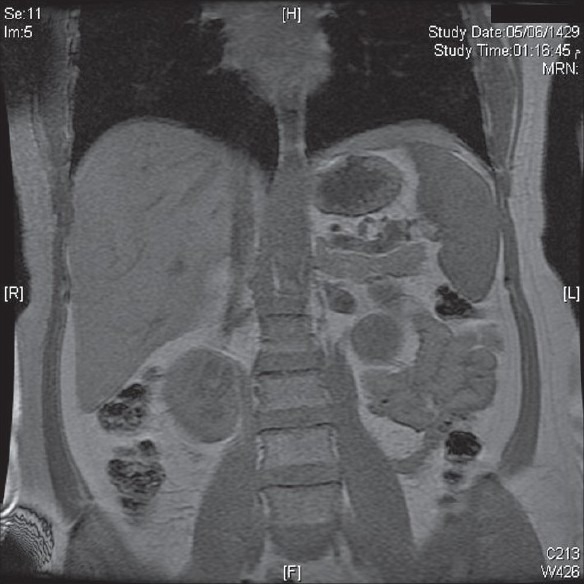
Coronal opposed - phase T1WI shows lack of signal drop from most of the mass except for minutes scattered foci indicating the presence of a minimal amount of microscopic fat

A 53-year-old female presented with complaints of intermittent, dull aching, vague abdominal pain of few months duration. The physical examination was unremarkable. A CT scan revealed a right adrenal mass measuring 6.5 × 5.2 cm. It was labelled as a fat containing mass, raising the possibility of lipoma, fat-rich adrenal adenoma or liposarcoma. The patient was subjected to multiplanar, multisequential magnetic resonance imaging (MRI) of the adrenal glands and pre- and post-gadolinium injections, to further evaluate this mass. The findings of were consistent with myelolipoma [Figure 1]. Laboratory investigations revealed the non-functioning nature of the adrenal mass, such as, urinary adrenaline 3 ug/24 hours (Ref: 27 ug/24 hours), noradrenaline 55 ug/24 hours (ref: 97 ug/24 hours) and Dopamine 454 ug/24 hours (ref: 500 ug/24 hours), in a total volume of 2600 ml of urine. Plasma biochemistry showed levels of adrenaline as 20 ug/L (ref: 84 ng/l), noradrenaline 163 ng/L (ref: 420 ng/l) and Dopamine 20 ng/L (ref: 85 ng/l). Urinary Vanillyl mandelic acid (VMA) was 3.0 mg/24 hours (ref: 6.8 mg/24 hours). Free Cortisol, metanephrines and normetanephrines were also within normal limits. CT-guided fine-needle aspiration cytology confirmed the diagnosis and the patient underwent laparoscopic right adrenalectomy with a smooth, postoperative recovery. Histopathology revealed myelolipoma.

### Case 2

A 43-year-old female, known to have hypertension, presented with right loin pain, occasional palpitations and increased flushing, for the last 20 months. The physical examination was unremarkable and an ultrasound (US) of the abdomen revealed a right adrenal mass lesion. Blood and urine investigations showed the non-functioning nature of the adrenal mass. A contrast CT revealed a non-enhancing mass lesion, arising from the lateral limb of the right adrenal gland, measuring 7 × 5.4 cm, consistent with fat, and further characterisation by a multiplanar MRI with a gadolinium injection showed a 7.2 × 5.7 × 6.1 cm mass arising from the lateral limb of the right adrenal gland, composed mainly of fat. Laboratory investigations revealed the non-functioning nature of the mass, such as, adrenaline 20 ng/L, noradrenaline 217 ng/L and plasma cortisol 53.81 nmol/l. Free cortisol in 24 hours collected urine (total volume 1700 ml) was 187.68 nmol/L. Urinary levels of adrenaline were 20 ug/24 hours (ref: 27 ug/24 hours), noradrenaline 24 ug/24 hours (ref: 97 ug/24 hours), Dopamine 377 ug/24 hours (ref: 500 ug/24 hours) and urinary VMA was 2.2 mg/24 hours (ref: 6.8 mg/24 hours). As there was no evidence of functioning pheochromocytoma, she was diagnosed as having essential hypertension and managed with medications and no metaiodobenzylguanidine (MIBG) scan was performed. A CT-guided fine-needle aspiration cytology confirmed the diagnosis of myelolipoma. Keeping in view the large size of the mass lesion and duration of symptoms, she underwent laparoscopic right adrenalectomy. The postoperative course was uneventful.

**Figure 2:**
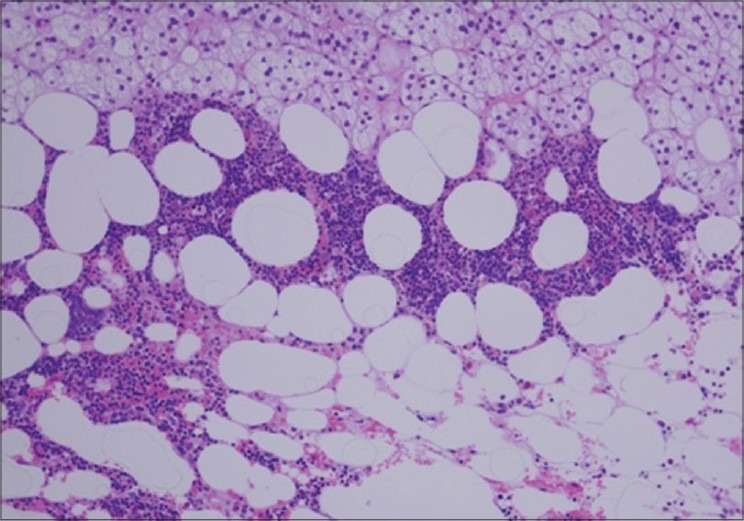
Histopathological picture showing fat lobules in the background of the stromal element (H&E, ×40)

Histopathology revealed myelolipoma with extramedullary haematopoiesis measuring 8.5 × 5 × 5 cm and weighing 80 grams [Figure 2].

## DISCUSSION

Myelolipomas are small, asymptomatic and non-functional in nature. The benign nature of these lesions has been established. Nevertheless, it remains unclear how this tumour actually develops. Although the pathogenesis of myelolipomas remains speculative, the most widely accepted theory is the existence of metaplasia of reticuloendothelial cells of blood capillaries in the adrenal glands, in response to stimuli, such as, necrosis, infection or stress.[5]

Myelolipomas in our patients were found with other medical conditions, such as, hypertension, diabetes mellitus and obesity. There is no proven link between these conditions and myelolipomas, yet elderly patients undergo imaging studies for other reasons, facilitating incidental diagnosis. Occasionally there are clinical symptoms such as abdominal pain or flank pain as in our patients. Spontaneous retroperitoneal haemorrhage in association with myelolipoma has been described.[6]

Histological examination of a myelolipoma shows adipocytes with interspersed haematopoietic elements, consisting of myeloid and erthyroid precursors, as well as, megakaryocytes.[6] When a suspicious mass is symptomatic or when diagnosis is not clear, surgical exploration becomes necessary. Traditionally the classic approach to this pathology has been open surgery.[6] Ever since its introduction by Gagner in 1992, laparoscopic adrenalectomy has become the standard of care for the treatment of functioning and non-functioning adrenal tumours. Many authors have found a decrease in perioperative morbidity and convalescence after this procedure when compared with open surgery.[7]

We conclude that adrenal myelolipomas are rare tumours. Patients with small, asymptomatic myelolipomas must be monitored clinically for symptoms and routine follow-up imaging studies appear unnecessary for such lesions. Surgical treatment must be reserved for selected patients with large and symptomatic lesions. Occasionally large silent tumours are excised, to prevent the occurrence of a rupture or intra-tumoural haemorrhage.
